# Secondary bacterial infections and antimicrobial resistance in COVID-19: comparative evaluation of pre-pandemic and pandemic-era, a retrospective single center study

**DOI:** 10.1186/s12941-021-00454-7

**Published:** 2021-08-05

**Authors:** Mustafa Karataş, Melike Yaşar-Duman, Alper Tünger, Feriha Çilli, Şöhret Aydemir, Volkan Özenci

**Affiliations:** 1 Faculty of Medicine, Ege University, İzmir, Turkey; 2 Department of Medical Microbiology, Faculty of Medicine, Ege University, İzmir, Turkey; 3 Division of Clinical Microbiology, Department of Laboratory Medicine, Karolinska Institutet, Stockholm, Sweden; 4 Department of Clinical Microbiology F 72, Karolinska Institutet, Karolinska University Hospital, Huddinge, 141 86 Stockholm, Sweden

**Keywords:** COVID-19, Secondary bacterial infections, Co-infections, Antimicrobial resistance

## Abstract

**Purpose:**

In this study, we aimed to evaluate the epidemiology and antimicrobial resistance (AMR) patterns of bacterial pathogens in COVID-19 patients and to compare the results with control groups from the pre-pandemic and pandemic era.

**Methods:**

Microbiological database records of all the COVID-19 diagnosed patients in the Ege University Hospital between March 15, 2020, and June 15, 2020, evaluated retrospectively. Patients who acquired secondary bacterial infections (SBIs) and bacterial co-infections were analyzed. Etiology and AMR data of the bacterial infections were collected. Results were also compared to control groups from pre-pandemic and pandemic era data.

**Results:**

In total, 4859 positive culture results from 3532 patients were analyzed. Fifty-two (3.59%) patients had 78 SBIs and 38 (2.62%) patients had 45 bacterial co-infections among 1447 COVID-19 patients. 22/85 (25.88%) patients died who had bacterial infections. The respiratory culture-positive sample rate was 39.02% among all culture-positive samples in the COVID-19 group. There was a significant decrease in extended-spectrum beta-lactamase (ESBL)-producing *Enterobacterales* (8.94%) compared to samples from the pre-pandemic (20.76%) and pandemic era (20.74%) (p = 0.001 for both comparisons). Interestingly, *Acinetobacter baumannii* was the main pathogen in the respiratory infections of COVID-19 patients (9.76%) and the rate was significantly higher than pre-pandemic (3.49%, p < 0.002) and pandemic era control groups (3.11%, p < 0.001).

**Conclusion:**

Due to the low frequency of SBIs reported during the ongoing pandemic, a more careful and targeted antimicrobial prescription should be taken. While patients with COVID-19 had lower levels of ESBL-producing *Enterobacterales, *the frequency of multidrug-resistant (MDR) *A. baumannii* is higher.

**Supplementary Information:**

The online version contains supplementary material available at 10.1186/s12941-021-00454-7.

## Introduction

Antimicrobial Resistance (AMR) is recognized as a public threat with constantly increasing urgency [1]. It is estimated that by the year 2050, 10 million deaths and an economic loss of $100 billion would be expected annually due to multidrug-resistant (MDR) (resistant to more than three or more antimicrobial categories) infections [2]. It is widely accepted that antimicrobial surveillance is crucial for tackling AMR globally [3]. The current pandemic, a consequence of severe acute respiratory syndrome coronavirus 2 (SARS-CoV-2) is associated with high mortality, morbidity, and healthcare-related costs [4]. Recent data shows that more than 175 million people have so far been infected and 3.7 million of them died [5]. Secondary bacterial infections (SBIs) result in higher mortality rates in patients with COVID-19 [6]. As previously reported in recent pandemics [7, 8], viral infections can also promote the development of bacterial invasive respiratory infections by impairing the immune response, enhancing the destruction of cells and tissue.

In the pandemic era, deaths and hospitalizations have been reported across all age groups, with a higher frequency in the elderly (> 60 years) patients [9]. SBIs have previously been reported in patients with COVID-19 [6, 10, 11]. It was recently shown that SBIs were observed in 15% of COVID-19 patients, while the SBIs were associated with 50% of all deaths [12]. Therefore, it is crucial to consider SBIs in order to improve the outcome of COVID-19. Consequently, patients with COVID-19 are treated with prophylactic broad-spectrum antibiotics in order to prevent and treat possible SBIs [13–16]. Recently, other approaches including phage therapeutics have been suggested as possible non-antibiotic treatment options [17].

Hygiene procedures such as hand hygiene, usage of disinfectants, personal protective equipment (PPE) are significantly changed due to the COVID-19 pandemic [18].

In environments where personnel and personal protective equipment (PPE) are insufficient, hygienic conditions may deteriorate, as well as increased use of PPE in interventions may prevent the increase of SBIs and the spread of common resistant bacterial strains. A previous study from Italy has suggested that lack of PPE, and lack of healthcare professionals associated with increased risk of spreading the carbapenem-resistant *Klebsiella pneumoniae* in the intensive care units (ICUs) [19].

It is important to evaluate the etiology and resistance patterns of SBIs as well as to compare the data with other patients before and during the pandemic. There is scarce data on SBI etiology and antimicrobial susceptibility data [10, 16]. However, hitherto published studies have not analyzed the changes in AMR in patients with COVID-19 and controls. In addition, there is no published data from developing countries on SBIs where high AMR is prevalent. The data is crucial for establishing effective antibiotic therapy as well as avoiding unnecessary treatment [20].

In this study, we aimed to evaluate the etiologies of SBIs and co-infections and AMR profiles of the bacterial pathogens causing these infections in patients with COVID-19. Also, our secondary aim was to reveal the differences in the AMR between patients with COVID-19 and other patients from the pre-pandemic and pandemic era.

## Methods

### Study design

This study was performed at Ege University Hospital, Izmir, with a total of 2426 patient beds. The study covers the data between December 15, 2019, and June 15, 2020, which can be considered as the preceding three months, and the first three months of the COVID-19 pandemic in Turkey.

### Study population

#### COVID-19 group

During the pandemic peak for SARS-CoV-2, real-time reverse transcription PCR (RT-PCR) assay positive and negative patients with symptoms consistent with COVID-19 were included. The RT-PCR assay negative patients had radiological imaging findings meeting the Ministry of Health's probable case [21] criteria for COVID-19 and therefore were included in the COVID-19 patient group (Additional file 2). SBIs are defined as infections occurring two days or more after patients were admitted to the hospital. Infections occurring not more than two days of hospitalization were defined as co-infections.

#### Control group

**Fig. 1:**
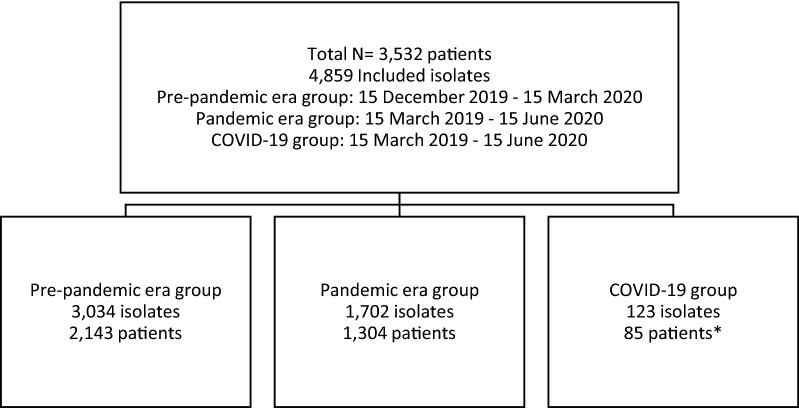
Flow chart of the study population.* A total of 1447 COVID-19 patients were studied and 85 had bacterial infections

This study included two control groups, i.e., one pre-pandemic era group with clinical microbiology culture results registered between December 15, 2019–March 15, 2020, and one pandemic era group with microbiology culture results registered between March 15, 2020–June 15, 2020, with no clinical and/or laboratory diagnosis of COVID-19 (Fig. 1).

### Laboratory methods

#### Bacterial culture

The investigation of bacterial pathogens in clinical samples was conducted with standard procedures as requested by the attending physician and was evaluated by microbiologists (Additional file 1). Identification and antimicrobial susceptibility testing of the isolates were performed using MALDI-TOF MS (Matrix-Assisted Laser Desorption/Ionization-Time of Flight Mass Spectrometry) (BioMérieux, France) and VITEK 2 (BioMérieux, France) automated systems, respectively. For viridans group streptococci, *Haemophilus* spp., *Corynebacterium striatum*, *Streptococcus pneumoniae,* and *Stenotrophomonas maltophilia*, the international Kirby Bauer disk diffusion method was used. All the susceptibility results were evaluated in accordance with the EUCAST criteria [22]. When imipenem or meropenem resistance was detected in *Enterobacterales* strains with the automated system, the result was confirmed by gradient test (BioMérieux, France). Antibiotic MIC values were confirmed by gradient test when isolates were determined resistant to vancomycin, teicoplanin, linezolid, tigecycline with VITEK 2 automated system. The VITEK 2 cefoxitin screen test was used to detect MRSA strains. Only one isolate per patient was studied.

#### SARS-CoV-2 RT-PCR

For PCR tests, DirectDetect™ SARS-CoV-2 Detection Kit (Coyote Bioscience Co, China) and Bio-Speedy SARS-COV2 (2019-nCoV) qPCR Detection Kit (Bioeksen R&D Technologies Ltd, Turkey) was used and according to the manufacturers’ recommendations. The PCR tests were performed with Qiagen Rotorgene Q-5 Plex-HRM Thermal Cycler (Qiagen, Belgium).

### Data collection

Blood, respiratory tract, urinary tract, and other samples such as gastrointestinal tract, tissue, normally sterile fluids cultures sent from patients were evaluated and susceptibility test results were collected from the microbiology laboratory database.

### Statistical analysis

Statistical analyses were performed using Microsoft Excel 2016 and IBM SPSS Statistics Version 18.0 (IBM Corp, Armonk, NY). Cross tables were created for categorical variables and chi-square analysis was performed. Categorical variables were shown as numbers and %, numerical variables as median (min., max.). *A. baumannii* changes between different groups were analyzed with two-tailed Fisher's exact test. Other comparisons (e.g., etiological changes, multidrug-resistant bacteria changes) were done with two-tailed Pearson's chi-squared test. A p value < 0.05 was considered statistically significant.

### Ethical approval

This study has been approved by the Ege University Medical Research Ethics Committee (20–9T/75) and Turkish Ministry of Health.

## Results

In total, 4859 culture-positive samples from 3532 patients were studied. The samples were collected between December 15, 2019, and June 15, 2020. The pre-pandemic era control group consists of 3034 samples from 2143 patients and the pandemic era control group included 1702 samples from 1304 patients. 1447 COVID-19 diagnosed patients’ data were evaluated separately, and 85 of them had 123 bacterial infections.

Of the 2143 patients in the pre-pandemic group, 1057 (49.32%) were female, 1086 (50.68%) were male, and the median age of these patients was 52 (0–99 years). Of the 1304 patients in the pandemic group, 630 (48.31%) were female, 674 (51.69%) were male, and the median age of these patients was 55 (0–100 years). 85 patients evaluated as COVID-19 patients, 45 (52.94%) were female and 40 (47.06%) were male and the median age of the patients was 61 (9 months-99 years). The mortality rate was 25.88% in COVID-19 patients who had been diagnosed with bacterial infections and 64 (38–96 years) was the median age among deaths.

### Pre-pandemic control group

**Fig. 2:**
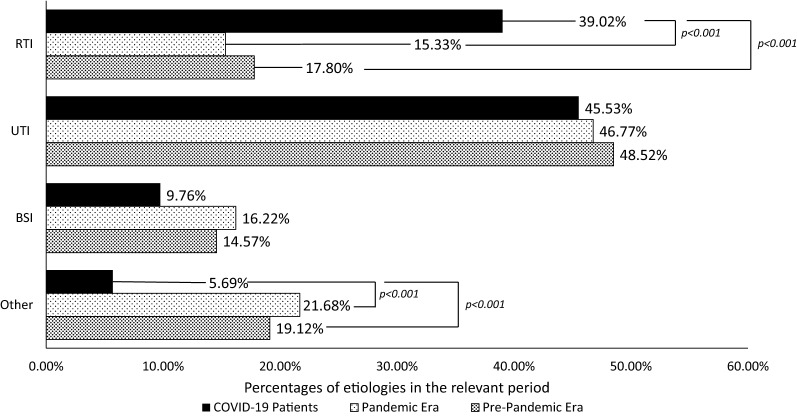
Etiology of the bacterial infections RTI: Respiratory Tract Infections UTI: Urinary Tract Infections BSI: Bloodstream Infections Other: Bacterial gastroenteritis, tissue infections, and infections in sterile body fluid

During the three months before the pandemic (December 15, 2019–March 15, 2020), microbiological data from 2143 patients were evaluated, and 3034 culture-positive samples were detected. Strains were mostly isolated from urine (1472, 48.52%), respiratory tract samples (e.g., sputum, bronchoalveolar lavage) (540, 17.32%), and blood samples (442, 14.57%). Other isolated samples came from different tissue samples such as wound swabs and sterile body fluids (455, 14.9%), stool (96, 3.1%), cerebrospinal fluid (29, 0.9%). The most common strains were *Escherichia coli* (766, 23.55%), *K. pneumoniae* (324, 11.56%), and *Pseudomonas aeruginosa* (254, 8.37%) (Fig. 2.). Extended-spectrum beta-lactamase ESBL-producing *Enterobacterales* were the most common (630, 20.76%) MDR bacteria among the strains isolated from these samples.

### Pandemic era control group

Microbiological data from 1304 patients were evaluated and 1702 culture-positive samples were included in the pandemic era control group. Strains were obtained from urine (796, 46.77%), blood (276, 16.22%), lower respiratory tract samples (261, 15.33%), and others (e.g., feces, tissue, and sterile fluid). The most common isolated strains were *E. coli* (447, 26.26%), *K. pneumoniae* (197, 11.57%), and *P. aeruginosa* (136, 7.99%) (Table 1). ESBL-producing *Enterobacterales* were the most common (353, 20.74%) type of MDR strain, followed by carbapenem-resistant *Enterobacterales* (CRE) (62, 3.64%).

**Table 1:** Bacterial strains detected in patients with COVID-19 and controls

Isolates	Pre-pandemic Era Control Group		Pandemic Era Control Group		COVID-19 patients	
	N^#^	%	N	%	N	%
*Escherichia coli*	766	25.25	447	26.26*	22	17.89*
*Klebsiella pneumoniae*	324	10.68	197	11.57	15	12.20
*Acinetobacter baumannii*	106	3.49***	53	3.11***	12	9.76***
*Corynebacterium striatum*	84	2.77	39	2.29	5	4.07
*Enterococcus faecium*	174	5.74	115	6.76	8	6.50
*Enterococcus faecalis*	201	6.62	122	7.17	9	7.32
*Pseudomonas aeruginosa*	254	8.37	136	7.99	8	6.50
*Staphylococcus aureus*	193	6.36	112	6.58	11	8.94
*Proteus mirabilis*	79	2.60	37	2.17	3	2.44
Other	853	28.11	444	26.09	30	24.39
*Total*	3034	100.00	1702	100.00	123	100.00

^#^N = numbers, * p < 0.05, ***p = 0.001

### COVID-19 patients

**Fig. 3:**
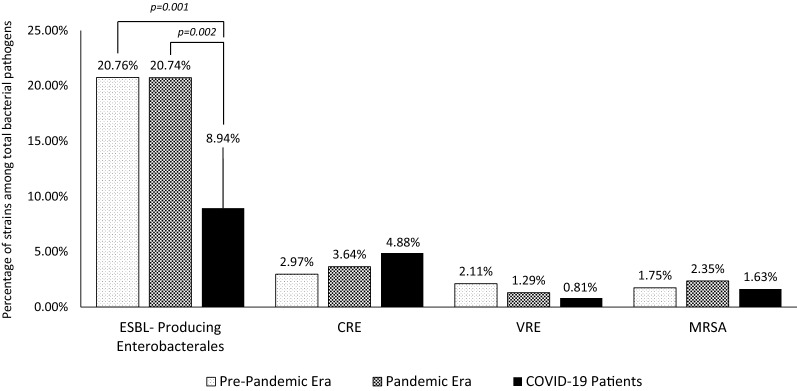
The most common multidrug-resistant isolates**.** ESBL: Extended-spectrum beta-lactamase CRE: Carbapenem-resistant *Enterobacterales* VRE: Vancomycin-resistant *Enterococcus* MRSA: Methicillin-resistant *Staphylococcus aureus*

The existence of bacterial infections in 1447 COVID-19 patients (both inpatient and outpatient) were evaluated. In total, 52/1447 (3.59%) patients had SBIs and 38/1447 (2.62%) had bacterial co-infections. Bacterial isolates were detected in 123 clinical samples. A secondary bacterial infection developed in five patients with bacterial co-infection during their follow-up. 28 (32.94%) patients had multiple bacterial, and 57 (67.05%) patients had mono-bacterial infections. The most common MDR organisms were ESBL-producing *Enterobacterales* (11, 8.94%) (Fig. 3).

In total, 78/123 (63.41%) bacterial strains were obtained two days after hospitalization of the patients and described as hospital-acquired infections while 45/123 (36.59%) were community-acquired infections. The most common bacteria was *A. baumannii* (10, 9.76%) among all respiratory tract samples (Table 2).

**Table 2:** Strains isolated from COVID-19 patients

	UTI	RTI	BSI	Other	Total
*Klebsiella pneumoniae*	9	4	2		15
*Enterococcus faecalis*	8		1		9
*Escherichia coli*	18	1	3		22
*Acinetobacter baumannii*	2	10			12
*Enterococcus faecium*	8				8
*Staphylococcus aureus*	1	7	1	2	11
*Pseudomonas aeruginosa*	2	5		1	8
*Corynebacterium striatum*		5			5
Other^a^	8	16	5	4	33
*Total*	56	48	12	7	123

*UTI* Urinary Tract Infections, *RTI* Respiratory Tract Infections, *BSI* Bloodstream Infections, *Other* Feces, tissue, and sterile fluid

^a^Four Stenotrophomonas* maltophilia*, three *Enterobacter cloacae*, three *Proteus mirabilis*, three *Streptococcus agalactiae*, two *Streptococcus pneumoniae,* two *Staphylococcus haemolyticus*, one *Staphylococcus epidermidis,* one *Corynebacterium* spp, one *Corynebacterium pseudodiphtheriticum,* one *Corynebacterium propinquum,* one *Campylobacter coli,* one *Acinetobacter junii,* one *Elizabethkingia meningoseptica,* one *Haemophilus influenzae* non-type B, one *Morganella morganii,* one *Pseudomonas stutzeri,* one *Pseudomonas putida,* one *Salmonella enterica* subsp. *enterica,* one *Streptococcus pyogenes.* All the strains and bacterial profiles of patients were shown in Additional file 3

## Discussion

Viral respiratory infections have been associated with an increased risk of bacterial infections. During the 2009 H1N1 outbreak, within 72 h of intensive care unit (ICU) admission, 30.3% of cases had bacterial co-infection [23]. Pandemics and seasonal flu data suggest that bacterial infections can worsen viral diseases and causes severe outcomes. Patients receiving invasive mechanical ventilation in other SARS and MERS epidemics developed secondary infections and had higher mortality [24]**.**

In the present study, 2.62% of patients with COVID-19 had bacterial co-infections whereas 3.59% of them had secondary bacterial infections. In total, 22/85 (25.88%) COVID-19 patients with bacterial infections died. Recent studies from Turkey showed that the overall mortality rate in patients with COVID-19 is 4.5% [25, 26]. Therefore, it is reasonable to suggest that bacterial infections are related to higher mortality rates in patients with COVID-19. UTI was the most common infection type (45.5%) and followed by RTI (39.02%) which was significantly higher than the two control groups. Previous studies analysing SBIs in patients with COVID-19 are contradictory. He et al*.* showed that 50% of the patients with COVID-19 had a SBI or carried bacterial pathogens [27]. However, in a recent meta-analysis, it was reported that the SBI rate in COVID-19 patients was between 4.7 and 19.5% and was associated with an increased risk of severe course or fatal outcomes [28, 29]. The underlying reason for low bacterial infection rates in the present study is not known. It might be related to several factors including the severity of the disease in patients included prior antimicrobial therapy or stringent local hygiene protocols applied during the pandemic era [30]. The observation of 3.59% of SBI suggests that empiric antibiotic treatment may not be necessary for all COVID-19 patients, since proven bacterial infections are relatively rare.

The most common pathogens in the COVID-19 study group were *E. coli* (22, 17.89%), *K. pneumonieae* (15, 12.2%), *A. baumannii* (12, 9.76%), and *S. aureus* (11, 8.94%). Detection of *E. coli* (22, 17.89%) was significantly lower in patients with COVID-19, compared to the pandemic era control group (447, 26.26%) (p = 0.04). In contrast, detection of *A. baumannii* in patients with COVID-19 was higher than in the two control groups.

In lower respiratory tract infections, the most detected pathogens were Gram-negative bacteria (32, 66.66%), following by Gram-positive bacteria (16, 33.33%). In Gram-negative bacteria, the most common isolated strains were *A. baumannii* (10/32, 31.25%), followed by *P. aeruginosa* (5/32, 15.63%)*.* Among Gram-positive bacteria, *S. aureus* (7/16, 43.75%) was the most common isolated strain. Although the distribution of Gram-negative and Gram-positive bacteria were similar, a higher *A. baumannii* occurrence as observed in our COVID-19 study group and needs further evaluation with comprehensive clinical data. A recent study from China showed that the most common bacterial pathogens isolated from respiratory tract samples were Gram-negative bacteria (26, 65%), following by Gram-positive bacteria (14, 34.99%). In that study, the most common bacterial pathogens encompassed *K. pneumoniae* (n = 11), *E. faecium* (n = 9), followed by *A. baumannii* (n = 8) [6]. The underlying reason for discrepant results between the present study and the previous report might be associated with differences in colonisation of bacteria types between centers, patients’ clinical profile as well as administration of prophylactic antibiotics.

ESBL-producing *Enterobacterales* were significantly lower in COVID-19 compared to pre-pandemic (p = 0.001) and pandemic era control group (p = 0.002). In a recent study which is reported from Egypt, Gram-negative isolates were mostly ESBL- or carbapenemase-producers which differs from our study [31]. The distinction may be due to the difference in the drug resistance profile between countries and/or local hygiene measures taken for patients with COVID-19. MDR *A. baumannii* was the most common bacteria (9.76%) in respiratory tract samples (39.02%) from COVID-19 patients, and this rate was significantly higher than that in the pre-pandemic (3.49%) (p = 0.002) and pandemic (3.11%) (p = 0.001) era control groups. In another study from New Jersey, when COVID-19 cases surge, increased carbapenem-resistant *A. baumannii* (CRAB) counts were reported. CRABs were mostly observed among COVID-19 patients admitted to ICUs and receiving ventilation therapy [32]. In both cases, the increase in intervention needed for COVID-19 patients may have led to a deterioration in hygiene conditions and an increase in the spread of MDR *A. baumannii.*

The present study has some limitations. First, not all COVID-19 patients were confirmed by PCR to be SARS-CoV-2 positive, and the comprehensive clinical data and disease severity of these patients were not studied. Secondly, a limited number of patients were available in our single-center study. However, we followed the official guidelines for the diagnosis of COVID-19, and it is highly unlikely that the patients with typical COVID-19 radiological findings had other infections. The lack of clinical severity data might be important, but the study focuses on the description of the prevalence of microorganisms and AST results in patients with COVID-19 in general. In addition, the sample size covers a period of 3 months in the pandemic era. The study has the following strengths: First, unlike other studies, we compared both the prevalence of bacterial infections and antimicrobial resistance patterns in patients with COVID-19 and control groups. Also, this study is the first comparative study presented from Turkey which is a developing country with a general high MDR bacteria profile.

## Conclusion

In conclusion, the present study shows that SBIs and bacterial coinfections were low in COVID-19 patients; but when present, it causes severe outcomes and is related to mortality. With these results, the administration of empirical broad spectrum antimicrobials to COVID-19 patients should be evaluated more carefully as the excessive use of antimicrobials could lead to a surge of antimicrobial resistance [33]. Also, while it was observed that COVID-19 patients had a lower risk of infected with ESBL-producing *Enterobacterales*; a significant increase in the MDR *A. baumannii* rates and increase of respiratory tract samples was observed and needs further evaluation. The present study will have an impact on diagnosis, possible treatments, and evaluating the existing sanitation measures in the hospitals. Further studies with larger sample size and detailed clinical data are warranted.

## Supplementary Information

**Additional file 1: Table S1.** Media used and incubation times according to sample types.

**Additional file 2.** Antimicrobial susceptibility patterns.

**Additional file 3: Table S1.** Detailed information on microorganisms and focus of infection isolated from patients with COVID-19.

## Data Availability

Authors can confirm that all relevant data are included in the article and/or its additional information files.
