# Supplementary material for: Secondary bacterial infections and antimicrobial resistance in COVID-19: comparative evaluation of pre-pandemic and pandemic-era, a retrospective single center study
Source: Ann Clin Microbiol Antimicrob. 2021 Aug 5;20:51. doi: 10.1186/s12941-021-00454-7 (PMC8340813; doi:10.1186/s12941-021-00454-7)
Supplement: Supplementary file 1 — Additional file 1: Table S1. Media used and incubation times according to sample types. [file 12941_2021_454_MOESM1_ESM.docx]

**Secondary Bacterial Infections and Antimicrobial Resistance in COVID-19: Comparative Evaluation of Pre-Pandemic and Pandemic-Era, A Retrospective Single Center Study.**

Mustafa Karataş^1^, Melike Yaşar-Duman^2^, Alper Tünger^2^, Feriha Çilli^2^, Şöhret Aydemir^2,¤^, Volkan Özenci^3,4,¤,*^

**Author Affiliations**

^1^Faculty of Medicine, Ege University, İzmir, Turkey

^2^Department of Medical Microbiology, Faculty of Medicine, Ege University, İzmir, Turkey

^3^Division of Clinical Microbiology, Department of Laboratory Medicine, Karolinska Institutet, Stockholm, Sweden

^4^Department of Clinical Microbiology F 72, Karolinska Institutet, Karolinska University Hospital, Huddinge, SE 141 86 Stockholm, Sweden

^¤^These authors contributed equally.

*Corresponding Author:

Volkan Ozenci, M.D., Ph.D. (volkan.ozenci@sll.se)

Associate Professor, Senior Consultant Physician

Clinical Microbiology F72

Karolinska University Hospital, Huddinge

S-14186 Stockholm

Sweden

**Supplementary Information 1. Patients’ Evaluation Criteria, Media used and incubation times according to sample types.**

**Evaluation Criteria of the COVID-19 Patients**

**COVID-19 Patients**

COVID-19 patients are individuals who have identified as “Probable case” according to Turkey Ministry of Health **and** other diagnoses are excluded with relevant tests (i.e., respiratory identifications panels including other viral pathogens, clinical manifestations) **and** treated with drugs (i.e., Hydroxychloroquine, Favipiravir together) for COVID-19 **and/or** confirmed with real-time reverse transcription PCR (RT-PCR) assay positive. Probable case criteria can be found in the [21] reference.

**Table S1. Media used and incubation times according to sample types.**

| Sample type | Culture | Incubation time |
| --- | --- | --- |
| Bloodstream samples | Aerobic, anaerobic, and pediatric bottles of the VersaTREK system used. Bottles were incubated in the VersaTREK^TM^ (TREK Diagnostic Systems, Cleveland, Ohio) blood culture system.  When bottles signaled positive, subcultures were made to 5% Sheep Blood agar (BioMérieux, France) and Eosin Methylene Blue (EMB) (BioMérieux, France) agar. | Bottles were incubated until they signaled positive or for a maximum five days (For *Brucella spp.* suspected cases a maximum of seven days).  Subcultures incubated for 24-48 hours |
| Urinary tract samples | By quantitative culture technique, 5% Sheep Blood agar and Eosin Methylene Blue agar was used. | Incubated for 24-48 hours |
| Sterile fluid samples | Direct microscobic examination (Fast Read 102®, Biosigma, Italy) was done.  Chocolate agar PolyViteX (BioMérieux, France), 5% Sheep Blood agar and Eosin Methylene Blue agar was used. | Incubated for 24-48 hours |
| Stool samples | First microscopic examination (for leukocyte and erythrocyte)  GN broth was used for the selective enrichment of Salmonella and Shigella  Methylene Blue agar and Hektoen agar (BioMérieux, France), Sorbitol MacConkey agar (BioMérieux, France) and Columbia agar+5% sheep blood (BioMérieux, France) was used | The samples were incubated for 4-6 hours in GN broth.  Incubated for 24-48 hours |
| Respiratory tract samples | Gram staining smear prepared and evaluated.  Chocolate agar PolyViteX (BioMérieux, France), 5% Sheep Blood agar and Eosin Methylene Blue agar was used. | Incubated for 24-48 hours |
