# Supplementary material for: Secondary bacterial infections and antimicrobial resistance in COVID-19: comparative evaluation of pre-pandemic and pandemic-era, a retrospective single center study
Source: Ann Clin Microbiol Antimicrob. 2021 Aug 5;20:51. doi: 10.1186/s12941-021-00454-7 (PMC8340813; doi:10.1186/s12941-021-00454-7)
Supplement: Supplementary file 2 — Additional file 2. Antimicrobial susceptibility patterns. [file 12941_2021_454_MOESM2_ESM.docx]

**Secondary Bacterial Infections and Antimicrobial Resistance in COVID-19: Comparative Evaluation of Pre-Pandemic and Pandemic-Era, A Retrospective Single Center Study.**

Mustafa Karataş^1^, Melike Yaşar-Duman^2^, Alper Tünger^2^, Feriha Çilli^2^, Şöhret Aydemir^2,¤^, Volkan Özenci^3,4,¤,*^

**Author Affiliations**

^1^Faculty of Medicine, Ege University, İzmir, Turkey

^2^Department of Medical Microbiology, Faculty of Medicine, Ege University, İzmir, Turkey

^3^Division of Clinical Microbiology, Department of Laboratory Medicine, Karolinska Institutet, Stockholm, Sweden

^4^Department of Clinical Microbiology F 72, Karolinska Institutet, Karolinska University Hospital, Huddinge, SE 141 86 Stockholm, Sweden

^¤^These authors contributed equally.

*Corresponding Author:

Volkan Ozenci, M.D., Ph.D. (volkan.ozenci@sll.se)

Associate Professor, Senior Consultant Physician

Clinical Microbiology F72

Karolinska University Hospital, Huddinge

S-14186 Stockholm

Sweden

**Additional Information 2. Antimicrobial Susceptilibity Patterns.**

|  | *E. coli* | *K. pneumoniae* | *A. baumanii* | *S. aureus* | *E. faecalis* | *E. faecium* | *P. aeruginosa* |
| --- | --- | --- | --- | --- | --- | --- | --- |
| Amikacin | 699S 13R 53I | 236S 54R 18I | 39S 62R 5I |  |  |  | 195S 48R 11I |
| Amoxicillin / Clavulanic acid | 323S 439R | 84S 222R 1I |  |  |  |  |  |
| Ampicillin | 208S 555R | 307R |  |  | 195S 6R | 21S 146R |  |
| Ertapenem | 747S 10R 5I | 197S 108R |  |  |  |  |  |
| Daptomycin |  |  |  | 69S 2R |  |  |  |
| Fosfomycin | 576S 18R | 132S 52R |  |  |  |  |  |
| Gentamicin | 584S 179R 2I | 186S 122R | 18S 88R | 183S 10R |  |  | 186S 64R |
| High dose gentamicin resistance |  |  |  |  | 128S 47R | 70S 66R |  |
| Clindamycin |  |  |  | 155S 38R |  |  |  |
| Levofloxacin |  |  | 2S 100R |  |  |  |  |
| Linezolid |  |  |  | 193S | 201S | 167S 7I |  |
| Oxacillin |  |  |  | 127S 53R |  |  |  |
| Meropenem | 754S 5R 6I | 203S 109R 11I | 4S 102R |  |  |  | 183S 48R |
| Netilmicin |  |  | 17S 89R |  |  |  | 145S 103R |
| Nitrofurantoin | 579S 15R |  |  |  |  |  |  |
| Penicillin |  |  |  | 21S 172R |  |  |  |
| Piperacillin / Tazobactam | 603S 123R 12I | 102S 174R 19I |  |  |  |  | 166S 86R |
| Cefepime | 55S 121R | 34S 87R |  |  |  |  | 181S 71R |
| Cefixime | 181S 414R | 68S 115R |  |  |  |  |  |
| Cefoxitin | 607S 113R 43I | 168S 139R |  | 128S 52R |  |  |  |
| Cefoperazone/sulbactam |  |  | 12S 75R 18I |  |  |  |  |
| Ceftazidime |  |  |  |  |  |  | 197S 57R |
| Ceftriaxone | 238S 524R | 103S 203R |  |  |  |  |  |
| Cefuroxime | 233S 530R | 101S 206R |  |  |  |  |  |
| Cefuroxime axetil | 233S 530R | 101S 206R |  |  |  |  |  |
| Ciprofloxacin | 458S 271R 36I | 125S 157R 26I | 3S 102R | 193S 42R | 145S 72R | 43S 133R | 177S 103R |
| Teicoplanin |  |  |  | 190S 3R | 199S 1R | 145S 41R |  |
| Tetracycline |  |  |  | 174S 19R |  |  |  |
| Tigecycline | 172S 4I | 24S 5R 76I | 50S 40R 31I | 190S | 200S | 161S 5R |  |
| Tobramycin |  |  | 31S 71R |  |  |  | 201S 50R |
| Trimethoprim/sulfamethoxazole | 435S 329R | 153S 154R | 39S 66R | 189S 4R |  |  |  |
| Vancomycin |  |  |  | 193S | 200S 1R | 144S 41R |  |
| Imipenem | 594S 1R | 143S 44R 10I | 4S 100R |  |  |  | 176S 71R 5I |

**Table 1. Antimicrobial susceptibility testing patterns of pre-pandemic era control group.**

**Table 2. Antimicrobial susceptibility testing patterns of pandemic era control group.**

|  | *E. coli* | *K. pneumoniae* | *A. baumanii* | *S. aureus* | *E. faecalis* | *E. faecium* | *P. aeruginosa* |
| --- | --- | --- | --- | --- | --- | --- | --- |
| Amikacin | 399S 36I 12R | 152S 8I 30R | 12S 6I 35R |  |  |  | 105S 5I 26R |
| Amoxicillin / Clavulanic acid | 186S 261R | 63S 127R |  |  |  |  |  |
| Ampicillin | 138S 309R | 190R |  |  | 117S 5R | 13S 93R |  |
| Ertapenem | 438S 5R | 129S 59R |  |  |  |  |  |
| Daptomycin |  |  |  | 33S 2R |  |  |  |
| Fosfomycin | 347S 8R | 83S 32R |  |  |  |  |  |
| Gentamicin | 334S 2I 109R | 140S 2I 48R | 16S 37R | 105S 7R |  |  | 100S 1I 35R |
| High dose gentamicin resistance |  |  |  |  | 79S 29R | 46S 47R |  |
| Clindamycin |  |  |  | 81S 29R |  |  |  |
| Levofloxacin |  |  | 2S 48R |  |  |  |  |
| Linezolid |  |  |  |  | 121S 1R | 106S |  |
| Oxacillin |  |  |  | 68S 36R |  |  |  |
| Meropenem | 443S 4R | 136S 9I 52R | 4S 49R |  |  |  | 89S 14I 33R |
| Netilmicin |  |  | 13S 40R | 111S 1R |  |  | 82S 53R |
| Nitrofurantoin | 350S 5R | 68S 47R |  |  |  |  |  |
| Penicillin |  |  |  | 17S 95R |  |  |  |
| Piperacillin / Tazobactam | 352S 7I 74R | 74S 22I 91R |  |  |  |  | 82S 53R |
| Cefepime | 12S 32R | 12S 31R |  |  |  |  | 99S 37R |
| Cefixime | 133S 270R | 53S 94R |  |  |  |  |  |
| Cefoxitin | 373S 14I 57R | 113S 77R |  | 68S 35R |  |  |  |
| Cefoperazone/sulbactam |  |  | 3S 11I 38R |  |  |  |  |
| Ceftazidime |  |  |  |  |  |  | 109S 27R |
| Ceftriaxone | 145S 299R | 64S 126R |  |  |  |  |  |
| Cefuroxime | 141S 303R | 62S 128R |  |  |  |  |  |
| Cefuroxime axetil | 141S 303R | 62S 128R |  |  |  |  |  |
| Ciprofloxacin | 271S 21I 155R | 84S 14I 92R | 2S 51R | 90S 14R | 82S 27R | 22S 72R | 87S 49R |
| Teicoplanin |  |  |  | 112S | 122S | 96S |  |
| Tetracycline |  |  |  | 94S 18R |  |  |  |
| Tigecycline | 37S | 22S 5I 16R | 22S 29I 2R | 112S | 122S | 102S 4R |  |
| Tobramycin |  |  | 20S 31R |  |  |  | 114S 22R |
| Trimethoprim/sulfamethoxazole | 257S 189R | 100S 90R | 10S 43R | 107S 5R |  |  |  |
| Vancomycin |  |  |  | 111S | 122S | 96S 18R |  |
| Imipenem | 404S 2R | 108S 15I 31R | 4S 1I 48R |  |  |  | 88S 8I 40R |

**Table 3. Antimicrobial susceptibility testing patterns of COVID-19 patients.**

|  | *E. coli* | *K. pneumoniae* | *A. baumanii* | *S. aureus* | *E. faecalis* | *E. faecium* | *P. aeruginosa* |
| --- | --- | --- | --- | --- | --- | --- | --- |
| Amikacin | 4S | 2I 5R | 2I 10R |  |  |  | 2R |
| Amoxicillin / Clavulanic acid | 5S 6R | 7S 8R |  |  |  |  |  |
| Ampicillin | 14S 8R | 15R |  |  | 8S | 8R |  |
| Ertapenem | 6S 1R | 4S 7R |  |  |  |  |  |
| Daptomycin |  |  |  |  |  |  |  |
| Fosfomycin | 19S | 4S 5R |  |  |  |  |  |
| Gentamicin | 20S 2R | 14S 1R | 2S 10R | 11S |  |  | 6S 2R |
| High dose gentamicin resistance |  |  |  |  | 3S 4R | 3S 3R |  |
| Clindamycin |  |  |  | 9S 1R |  |  |  |
| Levofloxacin |  |  | 12R |  |  |  |  |
| Linezolid |  |  |  | 4S | 4S 1R | 8S |  |
| Oxacillin |  |  |  | 9S 2R |  |  |  |
| Meropenem | 7S | 5S 6R | 12R |  | 1S |  | 1S 2I 1R |
| Netilmicin |  |  | 1S 11R |  |  |  | 3R |
| Nitrofurantoin | 13S 1R | 5S 4R |  |  |  |  |  |
| Penicillin |  |  |  | 1S 10R |  |  |  |
| Piperacillin / Tazobactam | 8S 4R | 8S 1I 6R |  |  |  |  | 6S 2R |
| Cefepime |  |  |  |  |  |  | 2S 2R |
| Cefixime | 8S 7R | 3S 11R |  |  |  |  |  |
| Cefoxitin |  |  |  | 9S 2R |  |  |  |
| Cefoperazone/sulbactam |  |  | 1I 11R |  |  |  |  |
| Ceftazidime |  |  |  |  |  |  | 7S 1R |
| Ceftriaxone | 3S 8R | 3S 11R |  |  |  |  |  |
| Cefuroxime | 1S 9R | 5S 10R |  |  |  |  |  |
| Cefuroxime axetil | 3S 9R | 5S 10R |  |  |  |  |  |
| Ciprofloxacin | 14S 7R 1I | 4S 8R | 12R | 11S | 2S 7R | 5R | 5S 3R |
| Teicoplanin |  |  |  | 3S | 4S | 7S 1R |  |
| Tetracycline |  |  |  | 9S 2R |  |  |  |
| Tigecycline |  |  | 3S 8I 1R | 3S | 6S | 8S |  |
| Tobramycin |  |  | 2S 10R |  |  |  | 3S 2R |
| Trimethoprim/sulfamethoxazole | 16S 6R | 9S 5R |  | 12S |  |  |  |
| Vancomycin |  |  |  | 3S | 4S | 7S 1R |  |
| Imipenem | 7S | 4S 6R 1I | 12R |  |  |  | 1S 2I 1R |
