# Supplementary material for: Secondary bacterial infections and antimicrobial resistance in COVID-19: comparative evaluation of pre-pandemic and pandemic-era, a retrospective single center study
Source: Ann Clin Microbiol Antimicrob. 2021 Aug 5;20:51. doi: 10.1186/s12941-021-00454-7 (PMC8340813; doi:10.1186/s12941-021-00454-7)
Supplement: Supplementary file 3 — Additional file 3: Table S1. Detailed information on microorganisms and focus of infection isolated from patients with COVID-19. [file 12941_2021_454_MOESM3_ESM.docx]

**Additional file 3: Table S1**. Detailed information on microorganisms and focus of infection isolated from patients with COVID-19.

| **Patient** | **Strain** | **Isolated from** | **Classified as** |
| --- | --- | --- | --- |
| **P1** | *Staphylococcus haemolyticus* | Urine | UTI |
| **P2** | *Klebsiella pneumoniae* | Blood | BSI |
| **P3** | *Klebsiella pneumoniae* | Urine | UTI |
|  | *Klebsiella pneumoniae* | Blood | BSI |
| **P4** | *Stenotrophomonas maltophilia* | Sputum | RTI |
| **P5** | *Escherichia coli* | Urine | UTI |
| **P6** | *Pseudomonas aeruginosa* | Tracheal Aspirate | RTI |
| **P7** | *Staphylococcus aureus* | Tracheal Aspirate | RTI |
|  | *Acinetobacter baumannii* | Tracheal Aspirate | RTI |
| **P8** | *Acinetobacter baumannii* | Tracheal Aspirate | RTI |
|  | *Klebsiella pneumoniae* | Tracheal Aspirate | RTI |
| **P9** | *Staphylococcus aureus* | Bronchoalveolar Lavage | RTI |
|  | *Escherichia coli* | Urine | UTI |
| **P10** | *Enterobacter cloacae* | Tracheal Aspirate | RTI |
| **P11** | *Staphylococcus aureus* | Wound | Other |
| **P12** | *Escherichia coli* | Blood | BSI |
| **P13** | *Klebsiella pneumoniae* | Urine | UTI |
| **P14** | *Klebsiella pneumoniae* | Sputum | RTI |
|  | *Escherichia coli* | Sputum | RTI |
| **P15** | *Escherichia coli* | Blood | BSI |
| **P16** | *Pseudomonas aeruginosa* | Urine | UTI |
| **P17** | *Klebsiella pneumoniae* | Tracheal Aspirate | RTI |
|  | *Staphylococcus aureus* | Tracheal Aspirate | RTI |
|  | *Pseudomonas aeruginosa* | Tracheal Aspirate | RTI |
|  | *Acinetobacter baumannii* | Tracheal Aspirate | RTI |
| **P18** | *Enterobacter cloacae* subsp. *cloacae* | Tracheal Aspirate | RTI |
|  | *Acinetobacter baumannii* | Sputum | RTI |
| **P19** | *Acinetobacter baumannii* | Tracheal Aspirate | RTI |
|  | *Pseudomonas aeruginosa* | Tracheal Aspirate | RTI |
| **P20** | *Enterococcus faecium* | Urine | UTI |
|  | *Escherichia coli* | Urine | UTI |
| **P21** | *Staphylococcus aureus* | Urine | UTI |
|  | *Staphylococcus aureus* | Blood | BSI |
|  | *Staphylococcus aureus* | Cerebrospinal Fluid | Other |
| **P22** | *Enterococcus faecium* | Urine | UTI |
| **P23** | *Proteus mirabilis* | Urine | UTI |
| **P24** | *Pseudomonas aeruginosa* | Sputum | RTI |
| **P25** | *Campylobacter coli* | Feces | Other |
| **P26** | *Acinetobacter baumannii* | Urine | UTI |
|  | *Acinetobacter baumannii* | Tracheal Aspirate | RTI |
| **P27** | *Klebsiella pneumoniae* | Urine | UTI |
| **P28** | *Escherichia coli* | Urine | UTI |
| **P29** | *Proteus mirabilis* | Blood | BSI |
| **P30** | *Streptococcus pneumoniae* | Sputum | RTI |
| **P31** | *Pseudomonas aeruginosa* | Urine | UTI |
| **P32** | *Corynebacterium striatum* | Bronchoscopic Aspiration | RTI |
| **P33** | *Enterococcus faecalis* | Blood | BSI |
| **P34** | *Escherichia coli* | Urine | UTI |
|  | *Enterococcus faecalis* | Urine | UTI |
| **P35** | *Enterococcus faecium* | Urine | UTI |
| **P36** | *Salmonella enterica* subsp*. enterica* | Feces | Other |
| **P37** | *Streptococcus agalactiae* | Urine | UTI |
| **P38** | *Escherichia coli* | Urine | UTI |
| **P39** | *Enterococcus faecium* | Urine | UTI |
| **P40** | *Enterococcus faecalis* | Urine | UTI |
|  | *Escherichia coli* | Urine | UTI |
| **P41** | *Klebsiella pneumoniae* | Urine | UTI |
| **P42** | *Enterococcus faecalis* | Urine | UTI |
|  | *Corynebacterium pseudodiphtheriticum* | Tracheal Aspirate | RTI |
|  | *Enterobacter aerogenes* | Tracheal Aspirate | RTI |
| **P43** | Coagulase Negative *Staphylococcus* | Wound | Other |
| **P44** | *Escherichia coli* | Urine | UTI |
|  | *Enterococcus faecalis* | Urine | UTI |
| **P45** | *Escherichia coli* | Urine | UTI |
|  | *Escherichia coli* | Blood | BSI |
|  | *Acinetobacter junii* | Tracheal Aspirate | RTI |
| **P46** | *Klebsiella pneumoniae* | Tracheal Aspirate | RTI |
| **P47** | *Escherichia coli* | Urine | UTI |
| **P48** | *Pseudomonas aeruginosa* | Tissue Biopsy | Other |
| **P49** | *Stenotrophomonas maltophilia* | Tracheal Aspirate | RTI |
| **P50** | *Enterobacter aerogenes* | Blood | BSI |
| **P51** | *Escherichia coli* | Urine | UTI |
|  | *Streptococcus pneumoniae* | Tracheal Aspirate | RTI |
| **P52** | *Staphylococcus aureus* | Tracheal Aspirate | RTI |
|  | *Corynebacterium striatum* | Tracheal Aspirate | RTI |
| **P53** | *Escherichia coli* | Urine | UTI |
| **P54** | *Enterococcus faecium* | Urine | UTI |
|  | *Streptococcus agalactiae* | Blood | BSI |
| **P55** | *Pseudomonas putida* | Urine | UTI |
| **P56** | *Acinetobacter baumannii* | Tracheal Aspirate | RTI |
| **P57** | *Acinetobacter baumannii* | Urine | UTI |
| **P58** | *Corynebacterium* spp. | Urine | UTI |
|  | *Enterococcus faecalis* | Urine | UTI |
| **P59** | *Enterococcus faecalis* | Urine | UTI |
| **P60** | *Enterobacter cloacae* | Tracheal Aspirate | RTI |
|  | *Stenotrophomonas maltophilia* | Tracheal Aspirate | RTI |
| **P61** | *Proteus mirabilis* | Urine | UTI |
| **P62** | *Escherichia coli* | Urine | UTI |
| **P63** | *Staphylococcus aureus* | Tracheal Aspirate | RTI |
| **P64** | *Enterococcus faecalis* | Urine | UTI |
|  | *Escherichia coli* | Urine | UTI |
| **P65** | *Staphylococcus aureus* | Sputum | RTI |
| **P66** | *Pseudomonas stutzeri* | Blood | BSI |
| **P67** | *Acinetobacter baumannii* | Tracheal Aspirate | RTI |
| **P68** | *Klebsiella pneumoniae* | Urine | UTI |
|  | *Corynebacterium striatum* | Tracheal Aspirate | RTI |
| **P69** | *Haemophilus influenzae* non-type B | Tracheal Aspirate | RTI |
|  | *Enterococcus faecium* | Urine | UTI |
| **P70** | *Escherichia coli* | Urine | UTI |
| **P71** | *Staphylococcus haemolyticus* | Sterile Body Fluid | Other |
| **P72** | *Escherichia coli* | Urine | UTI |
|  | *Stenotrophomonas maltophilia* | Sterile Body Fluid | UTI |
| **P73** | *Enterococcus faecium* | Urine | UTI |
| **P74** | *Streptococcus pyogenes* | Tracheal Aspirate | RTI |
| **P75** | *Klebsiella pneumoniae* | Urine | UTI |
| **P76** | *Escherichia coli* | Urine | UTI |
|  | *Enterococcus faecium* | Urine | UTI |
| **P77** | *Streptococcus agalactiae* | Urine | UTI |
| **P78** | *Enterococcus faecalis* | Urine | UTI |
| **P79** | *Escherichia coli* | Urine | UTI |
| **P80** | *Staphylococcus epidermidis* | Blood | BSI |
| **P81** | *Staphylococcus aureus* | Tracheal Aspirate | RTI |
|  | *Acinetobacter baumannii* | Tracheal Aspirate | RTI |
|  | *Morganella morganii* | Tracheal Aspirate | RTI |
|  | *Corynebacterium striatum* | Tracheal Aspirate | RTI |
|  | *Klebsiella pneumoniae* | Urine | UTI |
|  | *Elizabethkingia meningoseptica* | Tracheal Aspirate | RTI |
| **P82** | *Klebsiella pneumoniae* | Urine | UTI |
| **P83** | *Klebsiella pneumoniae* | Urine | UTI |
|  | *Acinetobacter baumannii* | Tracheal Aspirate | RTI |
|  | *Pseudomonas aeruginosa* | Tracheal Aspirate | RTI |
| **P84** | *Corynebacterium striatum* | Tracheal Aspirate | RTI |
| **P85** | *Corynebacterium propinquum* | Tracheal Aspirate | RTI |
